# Optimization of Ethanol Extraction Technology for Yujin Powder Using Response Surface Methodology with a Box–Behnken Design Based on Analytic Hierarchy Process–Criteria Importance through Intercriteria Correlation Weight Analysis and Its Safety Evaluation

**DOI:** 10.3390/molecules28248124

**Published:** 2023-12-15

**Authors:** Lidong Jiang, Wangdong Zhang, Wenbo Zhao, Yanzi Cai, Xue Qin, Baoshan Wang, Jiao Xue, Yanqiao Wen, Yanming Wei, Yongli Hua, Wanling Yao

**Affiliations:** College of Veterinary Medicine, Gansu Agricultural University, Lanzhou 730070, China; 15117259527@163.com (L.J.); zhangwd@gsau.edu.cn (W.Z.); 17899319801@163.com (W.Z.); 18794883564@163.com (Y.C.); 15734860282@163.com (X.Q.); wangbs2026@163.com (B.W.); 13208351581@163.com (J.X.); 13659465092@163.com (Y.W.); weiym@gsau.edu.cn (Y.W.); huayongli2004@163.com (Y.H.)

**Keywords:** Yujin powder, ethanol extracts, single factor test, AHP-CRITIC mixed weighting method, response surface method, safety test

## Abstract

Here, we aimed to optimize the ethanol extraction technology for Yujin powder (YJP) and evaluate its safety. The ultrasonic-assisted ethanol reflux extraction method refluxing was used to extract YJP. The parameters were optimized through a combination of single-factor and response surface methodology (RSM). The comprehensive Y value score calculated using the content of 13 active ingredients in YJP ethanolic extracts (YEEs) and the yield of the dry extract were used as measuring criteria. RSM with a Box–Behnken design using three factors and three levels was adopted to optimize the ethanol extraction technology for YJP. Finally, acute and subchronic toxicity tests were performed to evaluate its safety. The results revealed the best technological parameters: a liquid–material ratio of 24:1, an ethanol concentration of 69%, assistance of ultrasound (40 °C, 50 kHZ, 30 min), reflux time of 53 min, and reflux temperature of 50 °C. In acute toxicity tests, the maximum administration dosage in mice was 28.21 g/kg, which is higher than 10 times the clinical dosage. Adverse effects in the acute and subchronic toxicity tests were not observed. All clinical indexes were normal. In conclusion, the RSM based on AHP–CRITIC weight analysis could be used to optimize the ethanol extraction technology for YJP and YEEs prepared under the above conditions and ensure high safety.

## 1. Introduction

**Figure 1:**
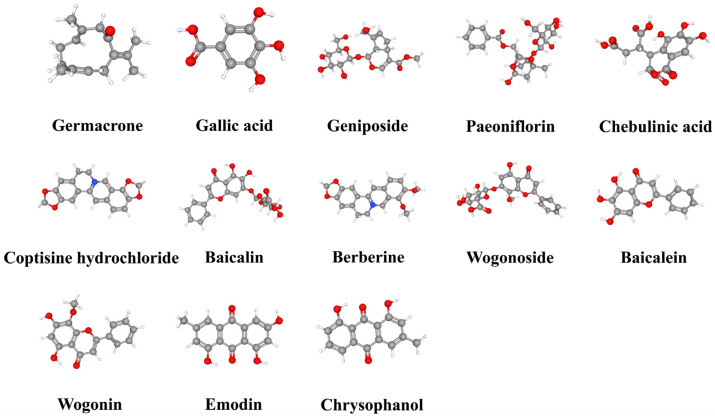
Three-dimensional depiction of 13 active components in Yujin powder. Red represents oxygen atoms, blue represents nitrogen atoms.

Yujin powder (YJP), from *Yuanheng Therapeutic Horse Collection*, is composed of Curcumae Radix, Chebulae Fructus, Scutellariae Radix, Radix Rhei Rhizome, Coptidis Rhizoma, Gardeniae Fructus, Paeoniae Radix Alba and Phellodendri Chinrnsis Cortex [1]. It has the effect of clearing heat and detoxicating and astringing intestines and stopping diarrhea [1]. In veterinary clinics, it is often used to treat dampness-heat diarrhea of horses, calf diarrhea and acute enteritis of cattle [2,3,4]. In addition, our previous research demonstrated that YJP had a good therapeutic effect on large intestine dampness-heat syndrome (LIDHS) [5,6,7]. In YJP, Curcumae Radix can activate blood circulation and dissolve stasis, relieve pain and alleviate jaundice symptoms [1]. Germacrone, a main ingredient of Curcumae Radix, has good anti-inflammatory [8], antioxidative [9] and antipyretic analgesic effets [10]. Coptidis Rhizoma, Scutellariae Radix, Phellodendri Chinensis Cortex and Gardeniae Fructus have the effect of clearing heat, drying dampness, purging fire and removing toxins [1]. Among them, coptisine, berberine, baicalin, baicalein, wogonoside, wogonin and geniposide are the main components which have anti-inflammatory and antioxidative effects [11,12,13,14,15]; coptisine and berberine have certain antibacterial activities and could protect the intestinal epithelial barrier [16]. Baicalin could also alleviate chronic gastritis effectively by inhibiting inflammatory factors [17]. Paeoniae Radix Alba and Chebulae Fructus can astringe Yin and intestines to stop diarrhea [1], and the main components in them (peoniflorin, chebulinic acid and gallic acid) could regulate immunity and intestinal flora and alleviate colitis [18,19]. Rhei Radix Rhizoma could clear blood heat and remove stagnation [1]; as the main effective components, emodin has strong antibacterial, anti-inflammatory and antioxidative effects and chrysophanol could inhibit vasoconstriction and promote blood clotting [20,21,22]. Therefore, we subsequently selected the content of the 13 active components, including germacrone, gallic acid, geniposide, paeoniflorin, chebulinic acid, coptisine hydrochloride, baicalin, berberine, wogonoside, baicalein, wogonin, emodin and chrysophanol (Figure 1) in YJP and the yield of the dry extract to assess the extraction effects of YJP.

At present, the powder and/or decoction of YJP are often used in the clinic. However, they have some disadvantages, such as less active ingredients, high volatility, difficult preservation, etc. [23]. Therefore, it is very necessary to develop some new dosage forms of YJP. Meanwhile, extracts need to be used as the raw material. The traditional extraction methods such as heat reflux extraction (HRE), Soxhlet extraction, distillation, etc., have some drawbacks, e.g., they are time consuming, have low extraction selectivity and efficiency, few active ingredients, high volatility, difficult preservation, etc. [24,25]. Therefore, new methods and assisted strategies need to be created to improve the extraction efficiency and purity. Ultrasound-assisted (UA) extraction is an efficient method for the extraction of herbal active ingredients. Relevant studies have shown that the extraction of flavonoids, saponins and salvianolic acid B using UA extraction was significantly higher compared to Soxhlet extraction, high-purity extraction and distillation [26,27]. The cavitation effects, mechanical effects and thermal effects of ultrasonic waves can fracture the cytoderm of medicinal material and then accelerate the release, diffusion and dissolution of material within cells. The extraction of medicinal materials with alcoholic solution could extract both the water-soluble and alcohol-soluble ingredients. And we found that UA extraction combined with ethanol refluxing extraction was the most effective of the extraction technologies from our previous exploration. Ultimately, the ultrasonic-assisted ethanol reflux extraction method was used to extract YJP [28].

Response surface methodology (RSM) has been widely used for optimizing the extraction technology of Traditional Chinese Medicine (TCM), such as in flavonoid extracts from *Paeonia lactiflora* seed peel, alkaloids from Rhizoma Coptidis, and so on [29,30]. The method has advantages including the high accuracy and reliability of the test parameters. Analytic hierarchy process (AHP) and criteria importance through intercriteria correlation (CRITIC) methods are often used for quality evaluation in TCM [31,32]. AHP means using mathematical logical thinking to analyze information from multiple targets. A pairwise comparison discriminant matrix is constructed, and the weight ratio of each piece of information to the preceding information is calculated [33]. CRITIC is used to comprehensively determine the objective weight of each indicator through the variability and conflict between indexes, and it can also determine the weight according to the size of the indicator variation [34]. The AHP–CRITIC method combines the advantages of the AHP and CRITIC, considering both subjective and objective factors. It is the most commonly used method of subjective and objective comprehensive weighting in TCM. Therefore, it is essential to develop the extraction technology of YJP based on Box–Behnken combined with an AHP-CRITIC design.

In the present study, the synthetic weighing method based on AHP and CRITIC was used to calculate the comprehensive scores. On the basis of single-factor experiments, RSM based on a Box–Behnken design was carried out to optimize the ethanol extraction technology parameters of YJP. In addition, the safety of YJP ethanolic extracts (YEEs) was evaluated. It is hoped that this study will provide a basis for the effective extraction of YEEs and lay a foundation for the development and utilization of YEEs.

## 2. Results

### 2.1. Method Validation of HPLC

#### 2.1.1. Linear Relationships

The calibration curves and linear ranges of germacrone, gallic acid, geniposide, paeoniflorin, chebulinic acid, coptisine hydrochloride, baicalin, berberine, wogonoside, baicalein, wogonin, emodin and chrysophanol are listed in Table 1.

#### 2.1.2. Precision, Stability and Repeatability

As shown in Table 2, Table 3 and Table 4, the precision, stability and repeatability RSD values of the thirteen standards were all < 2%, indicating that this method is suitable for the quantitative analysis of the 13 components.

#### 2.1.3. Sample Recovery Rate

Average recoveries ranged from 92.60% to 99.30%, and the RSD values were all < 2% for all thirteen compounds, indicating that the developed method was reliable and accurate enough for the measurement (Table 5).

### 2.2. AHP Weight

The normalized weight coefficients of germacrone, gallic acid, geniposide, paeoniflorin, chebulinic acid, coptisine hydrochloride, baicalin, berberine, wogonoside, baicalein, wogonin, emodin, and chrysophanol and the dry extract yield were 0.1541, 0.0467, 0.0897, 0.0467, 0.0467, 0.0467, 0.0897, 0.0897, 0.0897, 0.0897, 0.0897, 0.0467, 0.0467 and 0.0272, respectively (Table 6).

### 2.3. CRITIC Weight

The weight coefficients of germacrone, gallic acid, geniposide, paeoniflorin, chebulinic acid, coptisine hydrochloride, baicalin, berberine, wogonoside, baicalein, wogonin, emodin, and chrysophanol and the dry extract yield were 0.0711, 0.0776, 0.0916, 0.0767, 0.0524, 0.0607, 0.0584, 0.0611, 0.0654, 0.0697, 0.0653, 0.0726, 0.0751 and 0.1024, respectively (Table 7).

### 2.4. Weight Determination by AHP-CRITIC Mixed Weighting Method

The comprehensive weight coefficients of germacrone, gallic acid, geniposide, paeoniflorin, chebulinic acid, coptisine hydrochloride, baicalin, berberine, wogonoside, baicalein, wogonin, emodin, and chrysophanol and the dry extract yield were 0.1564, 0.0518, 0.1173, 0.0512, 0.0350, 0.0405, 0.0748, 0.0782, 0.0837, 0.0893, 0.0836, 0.0484, 0.0501 and 0.0398, respectively (Table 8).

### 2.5. Single-Factor Experiments

The comprehensive Y scores of 20 batches of the single-factor experiment were as follows: Y1 (liquid–material ratio 15:1) = 35.23%, Y2 (liquid–material ratio 20:1) = 45.55%, Y3 (liquid–material ratio 25:1) = 74.15%, Y4 (liquid–material ratio 30:1) = 52.80%, Y5 (reflux temperature 40 °C) = 39.81%, Y6 (reflux temperature 50 °C) = 47.27%, Y7 (reflux temperature 60 °C) = 52.74%, Y8 (reflux temperature 70 °C) = 45.61%, Y9 (ultrasonic intensity 40 kHZ) = 46.67%, Y10 (ultrasonic intensity 50 kHZ) = 50.94%, Y11 (ultrasonic intensity 60 kHZ) = 46.67%, Y12 (ultrasonic intensity 70 kHZ) = 42.43%, Y13 (reflux time 40 min) = 40.56%, Y14 (reflux time 50 min) = 60.99%, Y15 (reflux time 60 min) = 50.56%, Y16 (reflux time 70 min) = 47.05%, Y17 (ethanol concentration 50%) = 43.06%, Y18 (ethanol concentration 60%) = 51.18%, Y19 (ethanol concentration 70%) = 59.65%, Y20 (ethanol concentration 80%) = 45.68%.

**Figure 2:**
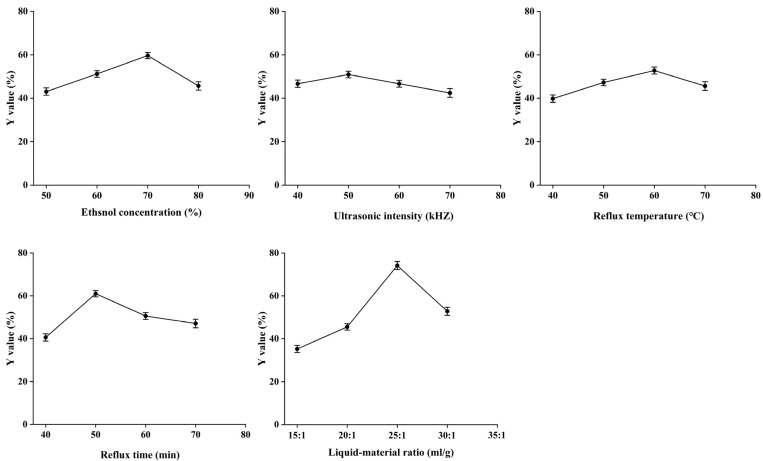
Y value of comprehensive score of five factors and four levels in single-factor experiment.

The results showed that Y value changed sightly with the increase in refluxing temperature and ultrasonic intensity, indicating that they had little influence on the extraction quality of YEEs. The other three factors significantly affected the extraction quality of YEEs. The three levels of ethanol concentration were 60%, 70%, and 80%, the refluxing times were 40 min, 50 min, and 60 min and the liquid−material ratios were 20:1, 25:1, and 30:1, respectively (Figure 2). 

### 2.6. Response Surface Experiment

#### 2.6.1. Model Establishment and Significance Test

Based on the results of single-factor experiments, a quadratic regression model was established using a three-factor-three-level full central composite experimental design based on RSM. The experimental design results are shown in Table 9. The quadratic regression equation is as follows:

Y = 89.28 − 4.67 × A + 0.72 × B − 3.44 × C − 0.15 × A × B − 1.21 × A × C − 0.055 × B × C − 11.46 × A^2^ − 4.48 × B^2^ − 5.67 × C^2^ (1)

Y is the comprehensive score; A, B and C represent the ethanol concentration, refluxing time and liquid–material ratio, respectively.

The model F = 181.33 and *p* < 0.0001 indicated that the model was highly significant. Lack-of-fit F = 3.40 and *p* = 0.1340 indicated that the lack-of-fit of the model was not significant and the experimental values greatly agreed with the predicted ones. R^2^ = 0.9957 indicated that the credibility of the model was good. To sum up, the model fully fitted the experimental data. The response value Y was related to the selected variables: ethanol concentration (A), refluxing time (B) and liquid–material ratio (C). As the F value is higher, the various factors have a more significant effect on the Y value. The F values of the ethanol concentration (A), refluxing time (B) and liquid–material ratio (C) were 253.96, 5.95 and 137.63, respectively. The influences of A, B and C on the Y value were highly significant (*p* < 0.05). The influence degree of each factor on the Y value showed the following order: ethanol concentration, liquid–material ratio, and refluxing time (Table 10).

#### 2.6.2. Validation of Response Surface Experiment

The optimal technology conditions were obtained as follows: ethanol concentration of 69%, liquid−material ratio of 24:1, and refluxing time of 53 min.

**Figure 3:**
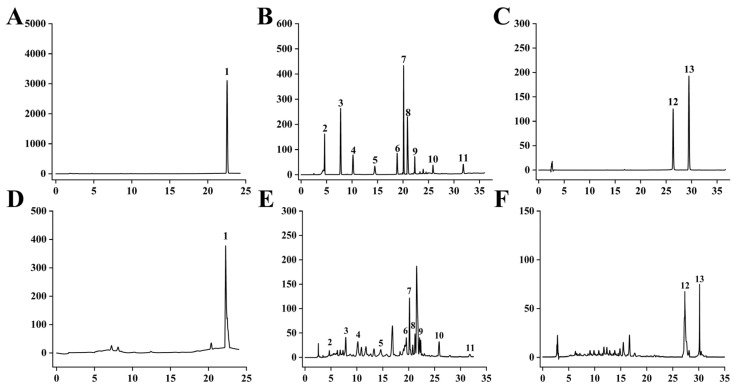
HPLC chromatograms of 13 components. (**A**–**C**) are the chromatograms of the standards. (**D**–**F**) are the chromatograms of the samples. 1—germacrone, 2—gallic acid, 3—geniposide, 4—paeoniflorin, 5—chebulinic acid, 6—coptisine hydrochloride, 7—baicalin, 8—berberine, 9—wogonoside, 10—baicalein, 11—wogonin, 12—emodin, 13—chrysophanol.

Under the above optimum conditions, we performed three parallel experiments with 24 g of crude drug each time, detected the content of 13 active ingredients in YJP, and calculated the yield of dry extract to calculate the comprehensive Y value score (Table 11, Figure 3). The weight of dry extract was 7.6944 ± 0.4080 g, and the yield of dry extract was 32.0613 ± 0.4989. Subsequently, an independent *t*-test was conducted using SPSS 26.0 software to compare the validation value with the predicted value. The result showed that the predicated value (90.0413) and validation value (90.0381) had no significant difference (*p* = 0.114 > 0.05), indicating that the predicated conditions of the model were identical to those of the actual situation and the model was successfully established. Therefore, the method was feasible and could be extensively enforced.

#### 2.6.3. Interaction of Various Factors

The response surface analysis was used to assess the interaction of various factors by Design Expert 8.0.6 software. The shape of the contour plot indicates whether the interaction among variables is significant, and ellipse denotes the strong interaction. The denser the contours were and the steeper the response surface graph was, the greater the influence of factors on the extract yield.

**Figure 4:**
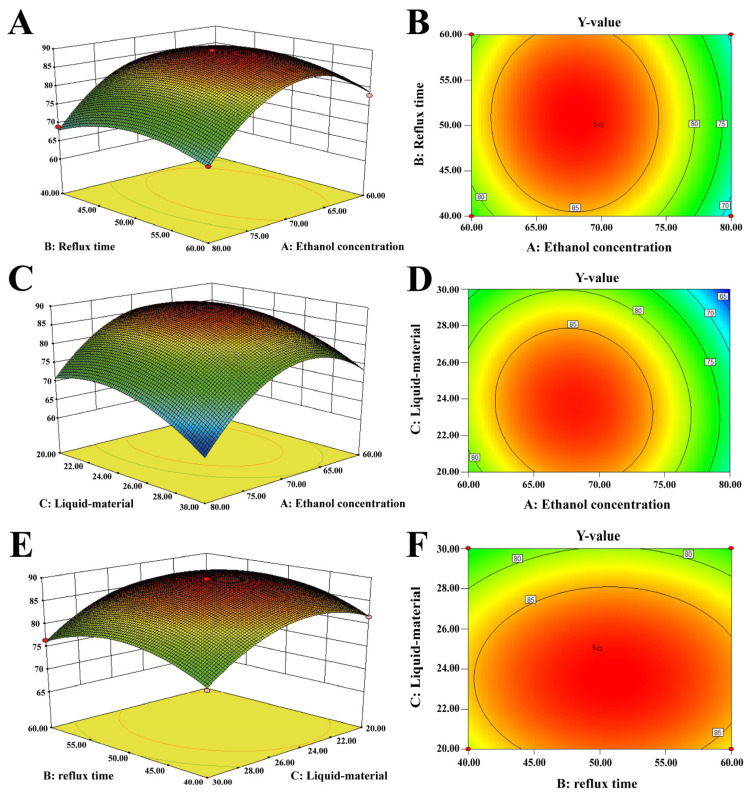
Interaction among various factors. (**A**,**C**,**E**) a three-dimensional response surface map; (**B**,**D**,**F**) a contour map.

As can be seen from Figure 4A, with the increase in ethanol concentration and reflux time, the Y value presented a trend of earlier increase and later decrease. As shown in Figure 4B, the contours along the ethanol concentration axis, which was elliptic, were denser than those along the refluxing time axis, indicating that the influence of ethanol concentration on the response values was more significant than that of the refluxing time and there was a strong and significant interaction between the two factors. Similarly, as can be seen from Figure 4C,D, with the increase in ethanol concentration and liquid–material ratio, the Y value presented a trend of earlier increase and later decrease, indicating that the influence of ethanol concentration on the response values was more significant than the liquid–material ratio. From Figure 4E,F, with the increase in liquid–material ratio and refluxing time, the Y value presented a trend of first increasing and later decreasing, indicating that the influence of the liquid–material ratio on the response values was more significant than the influence of the refluxing time.

To sum up, there were significant interactions among the ethanol concentration and refluxing time, ethanol concentration and liquid–material ratio, and refluxing time and liquid–material ratio. The influence on the comprehensive Y value score of YEEs showed the following order: ethanol concentration > liquid–material ratio > reflux time.

### 2.7. Acute Toxicity Test Results

#### 2.7.1. Pre-Experiment Results

After 7 days of administration, all the mice were active and in good spirits; appetite, stools and urine were all normal; breathing was even, and no deaths occurred. LD50 could not be detected, indicating that the YEEs were actually nontoxic. Therefore, we subsequently performed the maximum administration dosage test.

#### 2.7.2. Maximum Administration Dosage Test Results

The maximum administration dose was 28.21 g/kg (equal to 87.97 g/kg of crude drug) in one day, which was more than 10 times the dosage in the clinic. The mice in each group were active and in good spirits; appetites were all normal and no deaths occurred before and after drug administration.

### 2.8. Subchronic Toxicity Test Results

#### 2.8.1. Observation of Clinical Symptoms and Signs

After administration, the rats of the vehicle control group (VC group), high-dose YEEs group (HD-YEEs group), middle-dose YEEs group (MD-YEEs group) and low-dose YEEs group (LD-YEEs group) were active and in good spirits; appetite, stools and urine were all normal, breathing was even, and no deaths occurred. The weights showed an increasing trend for all rats, and the increase in the weight of the female rats was less than that of the male rats. There was no significant difference in the weights of male and female rats among the four groups (Table 12 and Table 13).

#### 2.8.2. Analysis of Organ Indices

As shown in Table 14 and Table 15, there was no significant difference in the heart, liver, spleen, lung, kidney, ovary and testis indices of male and female mice among the four groups.

#### 2.8.3. Detection Results of the Blood Routine and Blood Biochemistry

As shown in Table 16, Table 17, Table 18 and Table 19, there was no significant difference in the blood routine (WBC, LYM, HGB, RBC and PLT, etc.) and blood biochemistry indices (ALT, AST, ALP, Cr and BUN, etc.) of male and female mice among the four groups.

#### 2.8.4. Histopathological Change in Main Organs

**Figure 5:**
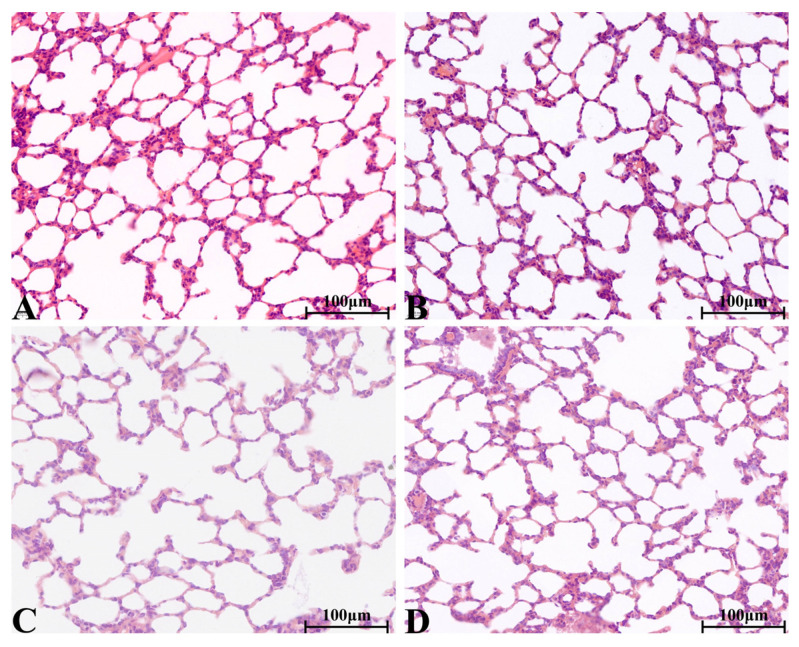
The H&E staining of serial parts of lung tissues in VC group (**A**), LD-YEEs group (**B**), MD-YEEs group (**C**) and HD-YEEs group (**D**). Original magnification, ×40. The scale bar represents 100 µm.

**Figure 6:**
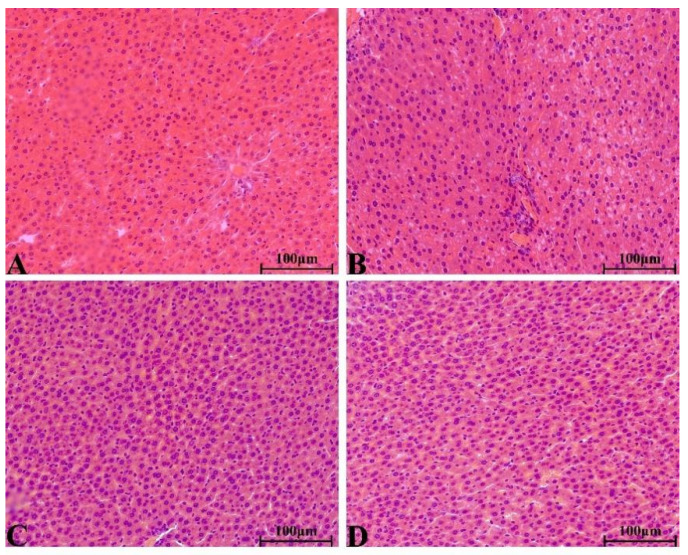
The H&E staining of serial parts of liver tissues in the VC group (**A**), LD-YEEs group (**B**), MD-YEEs group (**C**) and HD-YEEs group (**D**). Original magnification, ×40. The scale bar represents 100 µm.

**Figure 7:**
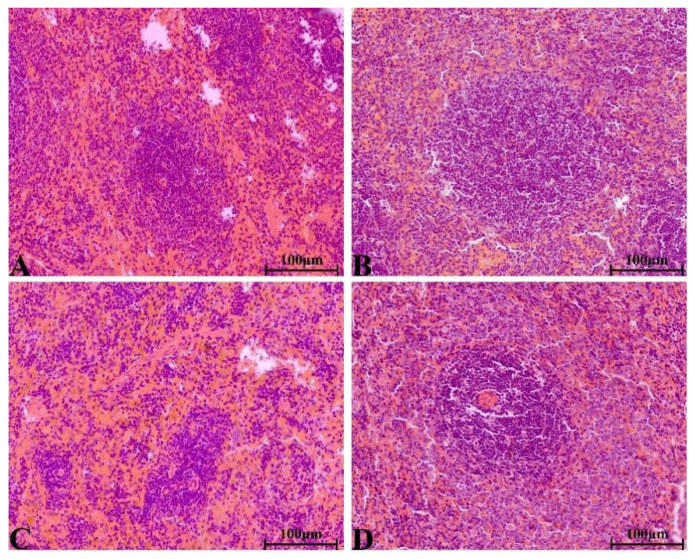
The H&E staining of serial parts of spleen tissues in the VC group (**A**), LD-YEEs group (**B**), MD-YEEs group (**C**) and HD-YEEs group (**D**). Original magnification, ×40. The scale bar represents 100 µm.

**Figure 8:**
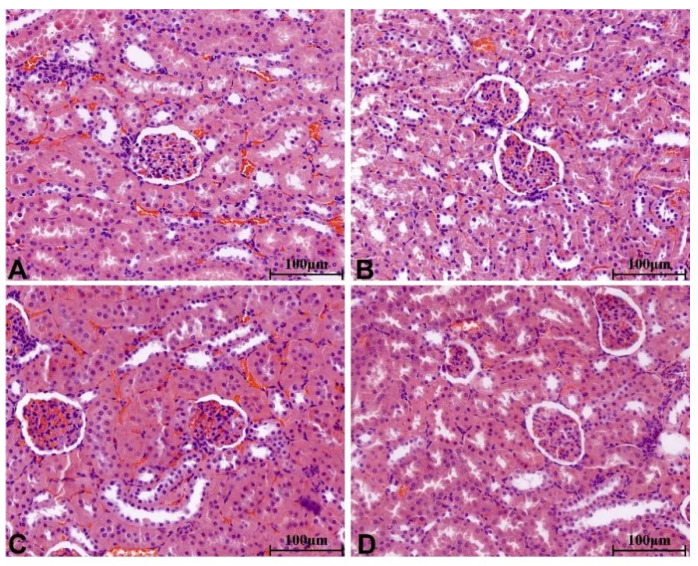
The H&E staining of serial parts of kidney tissues in the VC group (**A**), LD-YEEs group (**B**), MD-YEEs group (**C**) and HD-YEEs group (**D**). Original magnification, ×40. The scale bar represents 100 µm.

**Figure 9:**
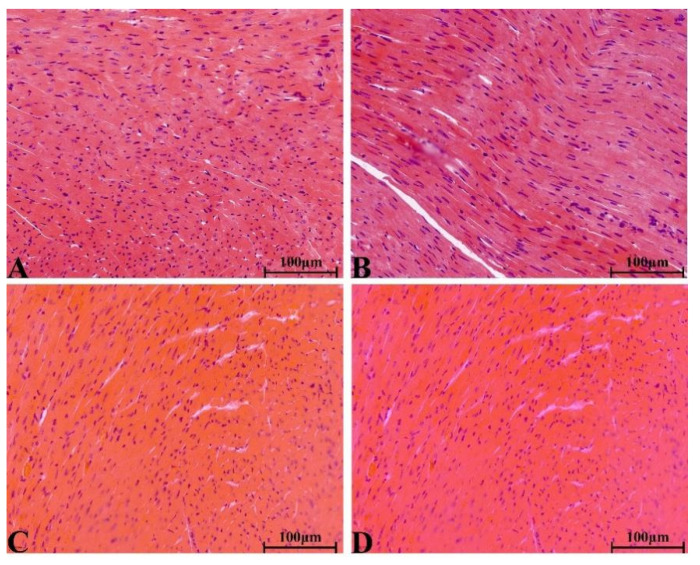
The H&E staining of serial parts of heart tissues in the VC group (**A**), LD-YEEs group (**B**), MD-YEEs group (**C**) and HD-YEEs group (**D**). Original magnification, ×40. The scale bar represents 100 µm.

**Figure 10:**
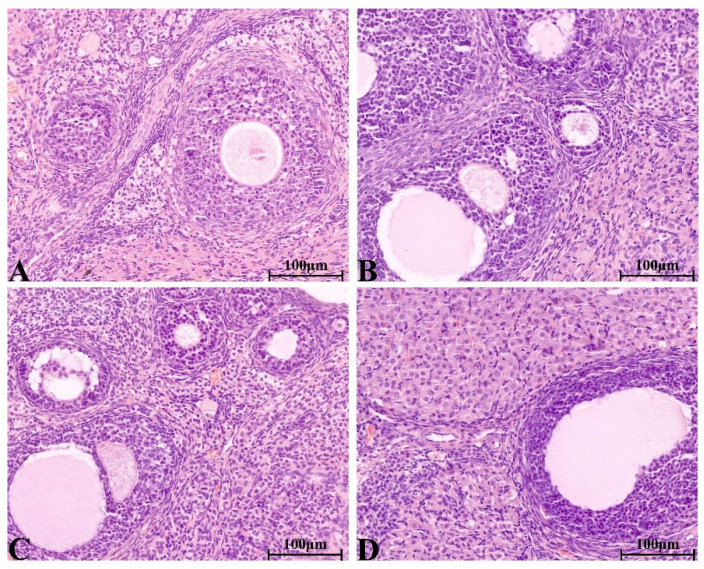
The H&E staining of serial parts of ovarian tissues in the VC group (**A**), LD-YEEs group (**B**), MD-YEEs group (**C**) and HD-YEEs group (**D**). Original magnification, ×40. The scale bar represents 100 µm.

**Figure 11:**
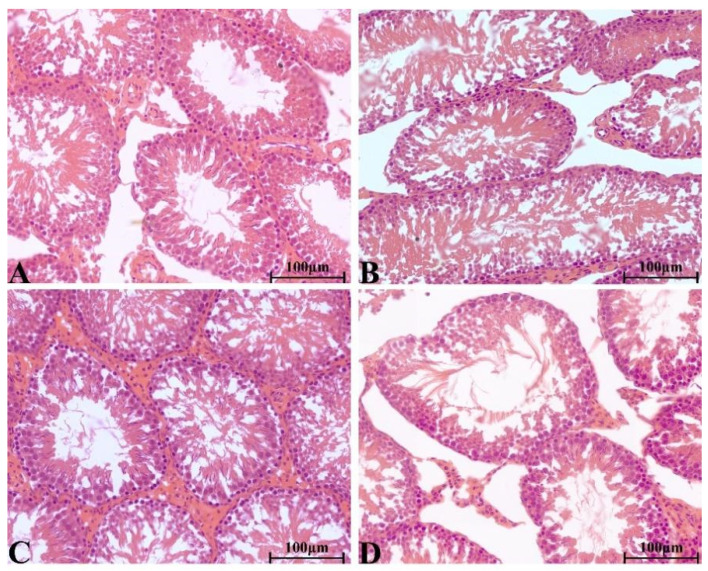
The H&E staining of serial parts of testes tissues in the VC group (**A**), LD-YEEs group (**B**), MD-YEEs group (**C**) and HD-YEEs group (**D**). Original magnification, ×40. The scale bar represents 100 µm.

There were no significant pathological changes in heart, liver, spleen, lung, kidney, ovary and testis of rats in each group. The structure of lung was clear, obvious atrophy; expansion and inflammatory cellular infiltrations were not observed (Figure 5). The structure of the liver and liver cells were regular and clear, the nucleus was located in the center of the cells, and the structure of the hepatic lobules was normal (Figure 6). The red pulp and white pulp of the spleen were sharply demarcated; the structure of the splenic corpuscle and central artery were clearly observed (Figure 7). The kidney and morphology of glomeruli were observed, expansion and atrophy were not observed, glomerular capsule cavity was smooth, and there was no exudation (Figure 8). The morphology of cardiomyocytes was normal; the myocardial fibers and cardiomyocytes were tightly connected and well arranged without atrophy, degeneration, edema and inflammatory infiltration (Figure 9). The morphological structure of the secondary oocyte in the ovary was normal, and the granular cell layer was well arranged without hyperemia and hemorrhage (Figure 10). The morphological structure of the testis was normal, the spermatocytes in the seminiferous tubules were evenly arranged, the mass of spermatids could be seen in the lumen and there was no thickening and obvious pathological changes in the interface membrane (Figure 11).

## 3. Discussion

The ethanol extraction technology of YJP was optimized by a single-factor test and RSM, which is a common method to optimize the extraction process of TCM. The comprehensive evaluation index was determined through the content of active ingredients and dry extract yield of YJP. The multi-objective components are transformed into single-objective optimization calculation by using a comprehensive evaluation method, which could not only merge together the comprehensive effects of all indices but also reflect the complexity of chemical composition and integrity and multi-targeted medicinal effects. It has been increasingly utilized in the extraction of Chinese herbal prescriptions in recent years. The key of the comprehensive evaluation method is to establish the weight coefficient of each index. In the present study, the synthetic weighing method based on the AHP and CRITIC was performed to calculate comprehensive scores. The compatibility of YJP and the objective factual data were both considered [35,36,37,38,39]. It could make the comprehensive scores more comprehensive, scientific and reasonable. The content of active ingredients in YJP was detected by HPLC, which is not restricted by the volatility and thermal stability of the samples, especially the compound with a high boiling point, macromolecular, strong polarity and poor heat stability. It is widely used in the analysis and detection of drug components due to the advantages of high sensitivity, fast analysis and high separation efficiency [40,41,42,43,44].

In the present study, the ultrasonic-assisted ethanol reflux extraction method was used to extract YJP. The ultrasonic intensity, ultrasonic time, ultrasonic temperature, ultrasonic frequency, reflux temperature, reflux time, liquid–material ratio and ethanol concentration were the main influential factors of ultrasonic-assisted ethanol reflux extraction, respectively [45,46,47,48,49,50,51]. The ultrasonic time and ultrasonic frequency should not be too long and high or the extraction yield of ineffective components increases, which is harmful for separation and purification; even the structures of active ingredients (flavonoids, alkaloids, glycosides) are damaged and hydrogen bonds in polysaccharides fracture, which might affect the biological activity [52]. Previous studies have shown that the ultrasonic time of 30 min and ultrasonic frequency of 300 W have a better effect on the extract of active ingredients such as flavonoids, polysaccharides, etc. [53,54]. Therefore, the ultrasonic time and ultrasonic frequency were set at 30 min and 300 W, respectively. Our previous experiment indicated that the optimum ultrasonic temperature was 40 °C. Therefore, we performed the single-factor experiment on only the following five factors: ultrasonic intensity, ethanol concentration, reflux temperature, reflux time, and liquid–material ratio. The results showed that the reflux temperature and ultrasonic intensity had less effect on the comprehensive Y score. Finally, the liquid–material ratio, ethanol concentration and reflux time were selected to perform an RSM experiment to obtain optimal extraction conditions. The interaction among the factors which affected the ethanol extraction technology of YJP was analyzed, and the impact degrees of several factors on the comprehensive Y score were in the following order: ethanol concentration > liquid–material ratio > reflux time. It was indicated that the selection of extraction solvent concentration played an important role in the ethanol extraction technology of YJP. Therefore, ethanol concentration had the greatest effect on the extraction rate of each component of YJP, which was followed closely by the liquid–material ratio and reflux time.

The YEEs is different from the traditional decoction and powder of YJP. Therefore, a safety evaluation must be performed before clinical application. We performed the acute toxicity and subchronic toxicity test on YEEs. The results of the toxicity test showed that YEEs were actually nontoxic and had no significant effect on the weight, main organ indices, blood routine, blood biochemistry and histopathological changes of various main organs. Therefore, it is safe and reliable under clinical doses. It is reported that each herb has high safety in YJP. The ethanol extract concentrate of the Curcumae Radix decoction pieces had no adverse effect on rats through subchronic toxicity [55]; the ethanol extract of Rhei Radix Rhizoma had minor liver and kidney damage only at the ultra-high dose [56]; the ethanol extract of Scutellariae Radix had low toxicity and was safe and reliable for clinical administration [57]; the ethanol extract of Gardeniae Fructus could cause liver and kidney damage in rats at a dosage of 0.14–0.56 g/kg, but it is reversible and much greater than the therapeutic dose [58]; the ethanol extracts of Coptidis Rhizoma, Phellodendri Chinensis Cortex, Chebulae Fructus and Paeoniae Radix Alba are usually used for anti-inflammatory, antibacterial and antioxidant research with little reports of toxicity [59,60,61,62]. To sum up, each of single herb of YJP has high safety, and the herbal ingredients are diluted compared with each single herb after formulation. Therefore, YEEs should have higher safety.

## 4. Materials and Methods

### 4.1. Materials and Reagents

#### 4.1.1. Experimental Drugs

Curcumae Radix, Coptidis Rhizoma, Scutellariae Radix, Phellodendri Chinensis Cortex, Gardeniae Fructus, Rhei Radix Rhizoma, Paeoniae Radix Alba and Chebulae Fructus (2:2:2:2:2:4:1:1) were purchased from the Yellow River medicine markets in Lanzhou City, Gansu Province, China. The mixture was crushed proportionately and passed through a 60-mesh screen.

#### 4.1.2. Experimental Reagents

Germacrone (batch number: YZ071922, CAS: 6902-91-6), gallic acid (batch number: YZ092122, CAS: 149-91-7), chebulinic acid (batch number: YZ0811222, CAS: 18942-26-2), geniposide (batch number: YZ081022, CAS: 24512-63-8), paeoniflorin (batch number: YZ032922, CAS: 23180-57-6), coptisine hydrochloride (batch number: YZ090423, CAS: 6020-18-4), berberine (batch number: YZ110920, CAS: 2086-83-1), baicalin (batch number: YZ051422, CAS: 21967-41-9), baicalein (batch number: YZ052421, CAS: 491-67-8), wogonoside (batch number: YZ053023, CAS: 51059-44-0), wogonin (batch number: YZ090220, CAS: 632-85-9), emodin (batch number: YZ080920, CAS: 518-82-1) and chrysophanol (batch number: YZDHF081020, CAS: 481-74-3) were purchased from Nanjing Yuanzhi Biotechnology Co., Ltd. (Nanjing, China). The purity of each reference component was determined to be above 98% by HPLC analysis. Chromatographic-grade methanol, acetonitrile and phosphoric acid were purchased from Sigma Aldrich (St. Louis, MO, USA). Anhydrous ethanol was obtained from the Tianjin Baishi Chemical Plant Co., Ltd. (Tianjin, China). Purified water was purchased from the Hangzhou Wahaha Group Co., Ltd. (Hangzhou, China).

#### 4.1.3. Animals

Kunming mice (half males and half females, 18–22 g) and SD rats (half males and half females, 160–180 g) were purchased from the Animal Center of the Lanzhou Veterinary Research Institute of the Chinese Academy of Agricultural Sciences (SCXK (Gan) 2020-0002). During the experiment, the mice were housed in a 12 h dark/light circulating environment of room temperature (25 ± 2 °C), relative humidity of 55 ± 5%, and were given free access to a standard diet and purified water. Animal welfare and experimental procedures were carried out in strict accordance with the “Guidelines for the Management and Use of Laboratory Animals” (Ministry of Science and Technology of China, 2006) and approved by the Animal Ethics Committee and the Animal Protection and Utilization Committee of Gansu Agricultural University.

### 4.2. Drug Preparation

The mixture of drugs listed in Section 4.1.1. was extracted by an ultrasonic-assisted ethanol reflux extraction method through different conditions including the ultrasonic intensity (30 kHZ, 40 kHZ, 50 kHZ, 60 kHZ), liquid–material ratio (15:1, 20:1, 25:1, 30:1), reflux temperature (40 °C, 50 °C, 60 °C, 70 °C), reflux time (40 min, 50 min, 60 min, 70 min) and ethanol concentration (50%, 60%, 70%, 80%). The mixture was firstly processed by ultrasound (Jining Tianhua ULTRASONIC Electronic Instrument Co. Ltd., Jining, China) (30 min, 40 °C) and then extracted by ethanol reflux twice. The filtrate obtained twice was mixed and concentrated by a rotary evaporator at 60 °C (Shanghai Yarong Biochemical Instrument Co., Ltd., Shanghai, China) and then freeze-dried by a vacuum freeze dryer (Beijing Boyikang Instrument Co., Ltd., Beijing, China). The dry extract was accurately weighed.

### 4.3. HPLC Detection

We use the method of high-performance liquid chromatography (HPLC) to detect the content of 13 active components of YEEs, which were used for the subsequent calculations of comprehensive Y value score [28].

#### 4.3.1. HPLC Chromatographic Condition

Agilent 1260 HPLC (Agilent Technologies, Santa, Clara, CA, USA) equipped with a Zorbax Eclipse Plus C18 column (4.6 × 250 mm, 5 μm) was used for HPLC analysis. The mobile phases were respectively composed of (A) aqueous phosphoric acid (0.1%, *v*/*v*) and (C) acetonitrile (gallic acid, geniposide, paeoniflorin, chebulinic acid, coptisine hydrochloride, berberine, baicalin, baicalein, wogonoside and wogonin), (A) aqueous acetic acid (10%, *v*/*v*) and (C) acetonitrile (emodin and chrysophanol), (A) ultrapure water and (C) acetonitrile (germacrone) at the flow rate of 1.0 mL/min. The following gradient elution conditions were used for the mobile phase A: 1–2 min, 95–85%, 2–13 min, 85–80%, 13–20 min, 80–62%, 20–30 min, 62–52%, 30–35 min, 52–41% (gallic acid, geniposide, paeoniflorin, chebulinic acid, coptisine hydrochloride, berberine, baicalin, baicalein, wogonoside and wogonin); 1–2 min, 95–85%, 2–10 min, 85–70%, 10–30 min, 70–40%, 30–40 min, 40–40% (emodin and chrysophanol); 0–15 min, 50–40%, 15–20 min, 40–15%, 20–25 min, 15–15%, 25–30 min, 15–5% (germacrone). The detection wavelengths were set at 250, 425 and 216 nm, respectively.

#### 4.3.2. Preparation of Standard Solutions

The appropriate amounts of the standards were weighed accurately, added to the same 10 mL volumetric flask and dissolved in 70% methanol. The mass concentrations of gallic acid, chebulinic acid, geniposide, paeoniflorin, coptisine hydrochloride, berberine, baicalin, baicalein, wogonoside, wogonin, emodin, chrysophanol and germacrone were 0.76, 0.5, 0.42, 0.1, 0.3, 0.7, 0.5, 0.25, 0.4, 0.57, 0.78, 0.48 and 0.889 mg/mL, respectively. Then, the mixed standard solution was diluted with methanol to obtain a series of solutions with different concentrations in order to establish the linear relationships. All the solutions were filtered using 0.22 μm microporous membranes before analysis and stored at 4 °C away from light.

#### 4.3.3. Method Validation with HPLC

The precision was validated by five replicate injections of mixed standard solutions under the optimized conditions. Stability was also tested by analyzing sample solutions at 0, 2, 4, 10 and 24 h. Five independent sample solutions of YEEs in parallel were prepared and analyzed for evaluating the repeatability. The sample recovery rate was conducted by adding thirteen accurately known quantities of the corresponding standards to a sample of YEEs that had previously been analyzed.

### 4.4. Weight and Calculation of Comprehensive Y Score

#### 4.4.1. Index for Selection

We consulted a lot of studies in the literature and summarized 13 active ingredients for treating the dampness-heat type of gastrointestinal diseases in YJP, including germacrone, gallic acid, geniposide, paeoniflorin, chebulinic acid, coptisine hydrochloride, berberine, baicalin, baicalein, wogonoside, wogonin, emodin and chrysophanol. The comprehensive score value calculated through the contents of the above active ingredients and the dry extract yield was used as the measuring index for the single-factor and RSM experiments.

#### 4.4.2. Weight Calculation by AHP

According to the compatible regularity and contribution of the monarch, minister, assistant and guide of YJP [63], the order of preference of 14 indicators was determined as follows: germacrone > berberine = baicalin = baicalein = wogonoside = wogonin = geniposide > chebulinic acid = gallic acid = paeoniflorin = emodin = chrysophanol = coptisine hydrochloride > dried extraction yield. According to this, we established the judgment priority matrix of paired comparison, and the relative scores of each index were obtained (Supplementary Table S1).

#### 4.4.3. Weigh calculation by CRITIC

The CRITIC method mainly embodies the objective information of the test data, and it divides the index weight into the contrast intensity (δi) between the indices and the conflict (Rij) between the indices [64].

(2) $$\mathrm{Rj}=\sum_{i=0}^{n}\left(1-\;\mathrm{rij}\;\right)=1/\bar{x},\;$$

δj is the standard deviation of the standardized column vector; Cj = δj, Rj = δj/$\bar{x}$, rij is the association coefficient of index i and j, i = 1, 2……m, $\bar{x}$ is the average value of the data in each row of the correlation matrix;

(3) $$\mathrm{Wj}=\mathrm{Cj}/\sum_{j=1}^{n}\mathrm{Cj}.$$

The data in Supplementary Table S2 are processed by linear interpolation (data index value = (measured value-minimum value)/(maximum value-minimum value) × 100%). After eliminating the unit dimension, the correlation coefficient matrix (A) was obtained through the process of SPSS 26 software. And the contrast intensity (δi), conflict (Rij), comprehensive weight (Ci) and weight (ωi) between the indicators were calculated according to the formula. The results are shown in Supplementary Tables S3 and S4.

#### 4.4.4. Weight Determination by AHP-CRITIC Mixed Weighting Method

The AHP-CRITIC mixed weighting method was used to evaluate weight (ω). The calculating formula is shown below:

ωsynthesis = ωAHPij × ωCRITICij/(∑ ωAHPij × ωCRITICij),(4)

where ωAHPij and ωCRITICij are the weight coefficients, respectively, by AHP and CRITIC [35,36,37].

#### 4.4.5. Calculation of Comprehensive Y Score

The contents of germacrone, gallic acid, geniposide, paeoniflorin, chebulinic acid, coptisine hydrochloride, berberine, baicalin, baicalein, wogonoside, wogonin, emodin and chrysophanol in the YEEs were detected by HPLC, and the extraction rate of each active ingredient and dry extract yield were calculated. According to the extraction rate of the above active ingredients in YJP and the dry extract yield, the AHP-CRITIC mixed weighting method was used to determine the weight coefficient and calculate the comprehensive Y score [38].

Synthesis score Y = [(germacrone content/maximum germacrone content) × ωsynthesis1 + (gallic acid content/maximum gallic acid content) × ωsynthesis2 + (geniposide content/maximum geniposide content) × ωsynthesis3 + (paeoniflorin content/maximum paeoniflorin content) × ωsynthesis4 + (chebulinic acid content/maximum chebulinic acid content) × ωsynthesis5 + (coptisine hydrochloride content/maximum coptisine hydrochloride content) × ωsynthesis6 + (baicalin content/maximum baicalin content) × ωsynthesis7 + (berberine hydrochloride content/maximum berberine hydrochloride content) × ωsynthesis8 + (wogonoside content/maximum wogonoside content) × ωsynthesis9 + (baicalein content/maximum baicalein content) × ωsynthesis10 + (wogonin content/maximum wogonin content) × ωsynthesis11 + (emodin content/maximum emodin content) × ωsynthesis12 + (chrysophanol content/maximum chrysophanol content) × ωsynthesis13 + (dry extract yield/maximum dry extract yield) × ωsynthesis14] × 100.

### 4.5. Single-Factor Experiment

The main influential factors of the extraction rate were ethanol concentration, reflux temperature, reflux time, the ratio of material to solvent and ultrasonic intensity from the literature. The single-factor test with five factors and four levels was designed: 50%, 60%, 70%, and 80% of ethanol concentration; 40 °C, 50 °C, 60 °C, and 70 °C of reflux temperature; 40 min, 50 min, 60 min, and 70 min of reflux time; 15:1, 20:1, 25:1, and 30:1 of the ratio of solvent to material; 30 kHZ, 40 kHZ, 50 kHZ, and 60 kHZ of ultrasonic intensity, respectively. According to the above extraction method, three parallel experiments were used to detect every index. And comprehensive Y score was used as the evaluation index.

### 4.6. Optimization of Ethanol Extraction Conditions of YJP by RSM

#### 4.6.1. Response Surface Experimental Design

The central composite design was performed by Design expert 8.0.6 software. The influence and interaction among ethanol concentration, reflux time and liquid–material ratio were investigated according to the Box–Behnken design principle; the matrix was generated, and the response surface model was analyzed (Table 20).

#### 4.6.2. Verification Experiments

The optimal conditions were determined by the analysis of Design expert 8.0.6 software. On the basis of optimizing the experimental results, the predicted optimal conditions were verified through three parallel experiments, and the comprehensive Y score was calculated and compared with the predicted value by an independent *t*-test.

### 4.7. Acute Toxicity Test

#### 4.7.1. Pre-Experiment

A total of 120 Kunming mice (18–22 g), half male and half female, were randomly divided into the vehicle control group (VC group), normal control group (NC group) and different dosages of YEEs groups (10 groups) with 10 mice in each group. All mice were fasted for 12 h but drank water freely before the experiment. The mice in the high-dose group were gavaged with YEEs with the maximum solubility in 0.5% carboxymethyl cellulose sodium (0.220 g/mL) and at the dose of 0.4 mL/10g body weight. The concentration of YEEs in the other 9 dosages groups were 0.176, 0.141, 0.113, 0.090, 0.072, 0.058, 0.047, 0.038 and 0.030 g/mL, respectively. All groups were gavaged 3 times every 6 h in one day. The mice in the NC and VC groups were gavaged with an equal volume of normal saline and 0.5% carboxymethyl cellulose sodium, respectively. The changes of the mental and active state, postures, hairs, stools, death, etc. of all mice were observed for 7 days, and the body weights were weighted every day during the experiment.

#### 4.7.2. Maximum Administration Dosage Test

The Lethal Dose50 (LD50) of YEEs was not detected, and its concentration could not be further increased. And the gavage volume of the mice does not exceed 0.4 mL/10g with no more than 3 times of gavage. According to the Technical Requirements for Traditional Chinese medicine New drug Pharmaco-toxicological Research, we performed the maximum administration dosage test. After acclimatization for 7 days, 40 Kunming mice (18–22 g), half male and half female, were randomly divided into VC and YEEs groups with 20 mice in each group. The mice in VC and YEEs groups were treated the same as the VC and high-dose groups, respectively, in Section 4.7.1. The observing and detecting indices were also the same as those in Section 4.7.1.

### 4.8. Subchronic Toxicity Test

#### 4.8.1. Animal Experiment

A total of 80 SD rats (160–180 g), half male and half female, were randomly divided into four groups with 20 rats in each group: the VC group, high-dose YEEs group (HD-YEEs group), middle-dose YEEs group (MD-YEEs group), and low-dose YEEs group (LD-YEEs group). After acclimatization for 7 days, the HD, MD, LD-YEEs groups were gavaged with YEEs (17.95, 7.82, 3.41 g/kg/day, respectively) once daily for 28 consecutive days, which was equal to 57.90 g/kg, 25.24 g/kg, and 11 g/kg of crude drug, respectively, and a corresponding volume of 0.5% carboxymethyl cellulose sodium in the VC group. The changes of the mental and active state, postures, hairs, appetites, stools, death, etc. of all rats were observed, and the mice were weighed every day during the experiment. After 28 days, all the rats were anesthetized intraperitoneally with 1% pentobarbital sodium. Blood samples were collected from abdominal aorta using EDTA-K2 and non-anticoagulant vacuum blood collection tubes. After that, the tissue samples of the heart, liver, spleen, lung, kidney, ovary and testicle were rapidly taken, weighed and then fixed in 10% neutral formalin. Finally, the organ indices were calculated (organ index = organ weight/body weight).

#### 4.8.2. Blood Routine Detection

The EDTA-K2 anticoagulated whole blood samples were performed blood routine detection using a Helner Exigo H400 Vet Auto Hematology Analyzer (Helner Corporation, Dalian, China). The indices mainly contained the white blood cell count (WBC), lymphocyte count (LYM), red blood cell count (RBC), hemoglobin (HGB), platelet (PLT), etc.

#### 4.8.3. Blood Biochemistry Detection

After 2–4 h standing at room temperature, the blood samples (from non-anticoagulant vacuum blood collection tubes) were centrifuged at 3000 rpm for 10 min. Serum samples were separated and stored at −80 °C for further use. The serum samples underwent performed biochemical detection using a Rayto Chemray 800 Auto Chemistry Analyzer (Rayto Corporation, Shenzhen, China). The indices mainly contained alanine transaminase (ALT), aspartate aminotransferase (AST), alkaline phosphatase (ALP), creatinine (Cr) and blood urea nitrogen (BUN).

#### 4.8.4. Histopathological Observation

After being fixed in 10% neutral formalin for more than 10 days, the standard pieces of tissue samples were embedded in paraffin, sliced at 4 µm, stained with hematoxylin and eosin (H&E) by a routine method, and observed by the light microscope.

### 4.9. Statistical Analysis

All data were expressed as mean ± SD (*n* = 10), which were tested by one-way analysis of variance (ANOVA) followed by the Duncan’s multiple range test. These were performed on IBM SPSS V.26.0 (SPSS Inc., Chicago, IL, USA). Significant differences were considered at *p* < 0.05.

## 5. Conclusions

In the present study, RSM based on a single-factor test was used for optimizing ethanol extraction technology of YJP with the comprehensive scores Y calculated through the APH-CRITIC method as the evaluation index. The ethanol extraction technology of YJP was optimized under the following conditions: ultrasound of 40 °C, 30 min, 50 kHZ; liquid–material ratio of 24:1, reflux time of 53 min, reflux temperature of 60 °C, and ethanol concentration of 69%. Under the above conditions, YEEs were actually nontoxic and had higher safety. The present study is of great significance to improve the utilization rate of YJP, and it could provide a basis for the application of new dosage forms of YJP.

## Figures and Tables

**Table 1:** Regression equation, linear range and correlation coefficient of 13 components in Yujin Powder.

Name	Regression Equation	Linear Range (μg/mL)	Correlation Coefficient R^2^
Germacrone	Y = 57355X + 126.29	27.80–222.25	0.9995
Gallic acid	Y = 16357X − 25.834	6.30–24.32	0.9993
Geniposide	Y = 21963X − 4.9886	26.24–100.00	0.9995
Paeoniflorin	Y = 5646.1X − 9.9892	20.82–80.00	0.9996
Chebulinic acid	Y = 16412X − 213.1	14.87–57.12	0.9996
Coptisine hydrochloride	Y = 59330X + 6.3053	3.12–12.00	0.9995
Baicalin	Y = 101568X − 202.95	26.03–100.00	0.9991
Berberine	Y = 51677X + 10.098	9.48–36.40	0.9990
Wogonoside	Y = 28001X − 30.971	4.17–16.00	0.9997
Baicalein	Y = 23263X + 53.541	2.60–100.00	0.9996
Wogonin	Y = 18631X − 2.4212	4.16–15.96	0.9992
Emodin	Y = 7890.3X + 9.258	7.50–28.80	0.9992
Chrysophanol	Y = 6005.5X − 23.322	4.87–18.72	0.9993

**Table 2:** Results of precision peak area RSD.

Name	Average	SD	RSD (%)
Germacrone	465.9	4.28	0.92
Gallic acid	1106.12	6.67	0.60
Geniposide	5999	38.86	0.65
Paeoniflorin	3964	31.37	0.73
Chebulinic acid	4914	12.8	0.36
Coptisine hydrochloride	3951	33.1	0.83
Baicalin	4459	25.5	0.57
Berberine	15,464	64.1	0.41
Wogonoside	1654	14.53	0.87
Baicalein	2654	19.1	0.72
Wogonin	848	5.36	0.63
Emodin	1014	7.62	0.75
Chrysophanol	119	0.77	0.65

**Table 3:** Results of stability peak area RSD.

Name	Average	SD	RSD (%)
Germacrone	388.46	4.76	1.23
Gallic acid	568.2	5.4	0.95
Geniposide	2406	8.4	0.35
Paeoniflorin	617	4.56	0.74
Chebulinic acid	711	3.23	0.45
Coptisine hydrochloride	846	3.65	0.55
Baicalin	2454	21.14	0.86
Berberine	2646	21.57	0.82
Wogonoside	560.6	3.98	0.71
Baicalein	524.5	4.87	0.93
Wogonin	670.5	5.14	0.77
Emodin	2008	7.85	0.39
Chrysophanol	343	3.16	0.92

**Table 4:** Results of repeatability peak area RSD.

Name	Average	SD	RSD (%)
Germacrone	309.24	3.01	0.97
Gallic acid	518.12	6.71	1.29
Geniposide	2245	15.74	0.70
Paeoniflorin	594	3.67	0.62
Chebulinic acid	664	7.84	1.18
Coptisine hydrochloride	762	3.19	0.42
Baicalin	2371	13.15	0.55
Berberine	2196	24.30	1.11
Wogonoside	524.5	4.63	0.88
Baicalein	497.31	4.11	0.82
Wogonin	641.3	4.29	0.67
Emodin	1974.9	6.35	0.32
Chrysophanol	290.1	1.47	0.51

**Table 5:** Determination results of sample recovery rate.

Name	Average	SD	RSD (%)	Rate of Recovery (%)
Germacrone	324.6	2.37	0.73	95.63
Gallic acid	901.06	8.47	0.94	93.71
Geniposide	1855.7	11.32	0.61	92.60
Paeoniflorin	1196.55	6.94	0.58	97.65
Chebulinic acid	2374.47	11.16	0.47	93.42
Coptisine hydrochloride	1929	9.45	0.49	94.28
Baicalin	2385.1	6.44	0.27	99.30
Berberine	10,112	15.45	0.15	92.88
Wogonoside	641.9	1.99	0.31	96.85
Baicalein	1109	8.98	0.81	95.52
Wogonin	829	1.16	0.14	96.44
Emodin	982	5.99	0.61	93.79
Chrysophanol	131.37	0.67	0.51	95.77

**Table 6:** AHP weight coefficient.

Name	Weight Coefficients
Germacrone	0.1541
Gallic acid	0.0467
Geniposide	0.0897
Paeoniflorin	0.0467
Chebulinic acid	0.0467
Coptisine hydrochloride	0.0467
Baicalin	0.0897
Berberine	0.0897
Wogonoside	0.0897
Baicalein	0.0897
Wogonin	0.0897
Emodin	0.0467
Chrysophanol	0.0467
Yield of dry extract	0.0272

**Table 7:** CRITIC weight coefficients.

Name	Weight Coefficients
Germacrone	0.0711
Gallic acid	0.0776
Geniposide	0.0916
Paeoniflorin	0.0767
Chebulinic acid	0.0524
Coptisine hydrochloride	0.0607
Baicalin	0.0584
Berberine	0.0611
Wogonoside	0.0654
Baicalein	0.0697
Wogonin	0.0653
Emodin	0.0726
Chrysophanol	0.0751
Yield of dry extract	0.1024

**Table 8:** AHP-CRITIC comprehensive weight coefficients.

Name	Comprehensive Weight Coefficients
Germacrone	0.1564
Gallic acid	0.0518
Geniposide	0.1173
Paeoniflorin	0.0512
Chebulinic acid	0.0350
Coptisine hydrochloride	0.0405
Baicalin	0.0748
Berberine	0.0782
Wogonoside	0.0837
Baicalein	0.0893
Wogonin	0.0836
Emodin	0.0484
Chrysophanol	0.0501
Yield of dry extract	0.0398

**Table 9:** Box–Behnken response surface test factor level, experiment design and results.

No.	(A) Ethanol Concentration%	(B) Reflux Time min	(C) Liquid–Material Ratio mL/g	Y Value%
1	80.00	50.00	30:1	62.4835
2	70.00	50.00	25:1	89.7089
3	70.00	50.00	25:1	88.5748
4	60.00	50.00	30:1	75.3015
5	70.00	60.00	20:1	83.8701
6	80.00	60.00	25:1	69.4049
7	70.00	40.00	20:1	81.6292
8	70.00	50.00	25:1	88.7082
9	70.00	60.00	30:1	76.5164
10	60.00	60.00	25:1	78.0018
11	80.00	50.00	20:1	71.4199
12	60.00	50.00	20:1	79.4086
13	70.00	50.00	25:1	89.6892
14	60.00	40.00	25:1	76.9670
15	80.00	40.00	25:1	68.9748
16	70.00	40.00	30:1	74.4967
17	70.00	50.00	25:1	89.7066

**Table 10:** Analysis of quadratic model variance table of response surface.

Project	Sum of Squares	Degree of Freedom	Mean Square	F Value	*p* Value	Significance
Modal	1123.35	9	124.82	181.33	<0.0001 **	Significant
A	174.81	1	174.81	253.96	<0.0001 **	
B	4.10	1	4.10	5.95	0.0448 *	
C	94.74	1	94.74	137.63	<0.0001 **	
AB	0.091	1	0.091	0.13	0.7263	
AC	5.83	1	5.83	8.47	0.0226 *	
BC	0.012	1	0.012	0.018	0.8977	
A2	552.74	1	552.74	803.02	<0.0001 **	
B2	84.61	1	84.61	122.93	<0.0001 **	
C2	135.20	1	135.20	196.42	<0.0001 **	
Residual error	4.82	7	0.69			
Misfit term	3.46	3	1.15	3.40	0.1340	Not significant
Pure error	1.36	4	0.34			
Total deviation	1128.17	16				

* indicated significant difference (*p* < 0.05); ** indicated extremely significant difference (*p* < 0.01).

**Table 11:** Content of each chemical composition in YEEs in validation experiment (mg/g).

Name	Content in YEEs
Germacrone	1.3160 ± 0.0055
Gallic acid	6.5340 ± 0.01078
Geniposide	17.7005 ± 0.0070
Paeoniflorin	89.6883 ± 0.0271
Chebulinic acid	17.5867 ± 0.0093
Coptisine hydrochloride	3.9456 ± 0.0023
Baicalin	14.6616 ± 0.0047
Berberine	98.2565 ± 0.0100
Wogonoside	5.0976 ± 0.0066
Baicalein	1.9298 ± 0.0020
Wogonin	2.8679 ± 0.0020
Emodin	1.0792 ± 0.0037
Chrysophanol	17.2449 ± 0.0096

**Table 12:** Body weight changes of male rats (x ± s).

Group	Initial Weight	First Week	Second Week	Third Week	Fourth Week
HD-YEEs group	209.71 ± 4.60	220.04 ± 3.86	243.87 ± 4.49	300.19 ± 10.42	315.15 ± 6.90
MD-YEEs group	205.69 ± 2.04	227.17 ± 2.80	258.02 ± 2.86	291.75 ± 2.39	320.42 ± 1.55
LD-YEEs group	210.97 ± 3.93	232.75 ± 4.83	264.19 ± 3.86	304.72 ± 4.54	332.90 ± 4.42
VC group	210.25 ± 4.15	233.61 ± 2.25	266.38 ± 2.87	302.68 ± 5.82	323.11 ± 8.64

Note, there was no significant difference among the four groups.

**Table 13:** Body weight changes of female rats (x ± s).

Group	Initial Weight	First Week	Second Week	Third Week	Fourth Week
HD-YEEs group	193.39 ± 5.23	196.33 ± 4.14	205.21 ± 3.28	219.45 ± 1.81	228.22 ± 2.68
MD-YEEs group	189.05 ± 2.95	206.18 ± 2.34	220.99 ± 1.78	229.96 ± 1.66	235.44 ± 2.82
LD-YEEs group	198.12 ± 1.77	202.13 ± 3.02	213.73 ± 7.42	238.49 ± 7.47	237.89 ± 11.26
VC group	196.74 ± 3.13	202.57 ± 3.29	211.59 ± 3.52	227.90 ± 9.04	231.23 ± 8.03

Note, there was no significant difference among the four groups.

**Table 14:** Effect of YEEs on organ indices of male rats (%).

Group	Heart	Liver	Spleen	LUNG	Kidney	Testis
HD-YEEs group	0.30 ± 0.01	2.65 ± 0.03	0.21 ± 0.01	0.43 ± 0.02	0.69 ± 0.03	1.02 ± 0.16
MD-YEEs group	0.30 ± 0.01	2.68 ± 0.16	0.21 ± 0.02	0.39 ± 0.01	0.66 ± 0.06	0.99 ± 0.10
LD-YEEs group	0.31 ± 0.01	2.66 ± 0.06	0.21 ± 0.01	0.41 ± 0.02	0.69 ± 0.02	0.99 ± 0.08
VC group	0.30 ± 0.01	2.56 ± 0.05	0.20 ± 0.01	0.42 ± 0.02	0.65 ± 0.02	0.91 ± 0.07

Note, there was no significant difference among the four groups.

**Table 15:** Effect of YEEs on organ indices of female rats (%).

Group	Heart	Liver	Spleen	Lung	Kidney	Ovary
HD-YEEs group	0.32 ± 0.02	2.45 ± 0.04	0.28 ± 0.02	0.57 ± 0.06	0.73 ± 0.02	0.32 ± 0.03
MD-YEEs group	0.32 ± 0.01	2.52 ± 0.06	0.25 ± 0.02	0.59 ± 0.07	0.73 ± 0.02	0.37 ± 0.01
LD-YEEs group	0.32 ± 0.02	2.79 ± 0.20	0.26 ± 0.01	0.50 ± 0.03	0.70 ± 0.10	0.33 ± 0.02
VC group	0.33 ± 0.01	2.67 ± 0.11	0.26 ± 0.02	0.54 ± 0.02	0.67 ± 0.07	0.34 ± 0.01

Note, there was no significant difference among the four groups.

**Table 16:** Effect of YEEs on blood routine of male rats (x ± s).

Group	WBC (10^9^/L)	LYM (10^9^/L)	HGB (10^9^/L)	RBC (10^12^/L)	PLT (10^9^/L)
HD-YEEs group	7.01 ± 0.11	5.41 ± 0.12	15.57 ± 0.16	7.20 ± 0.15	1126.80 ± 7.95
MD-YEEs group	7.44 ± 0.30	6.12 ± 0.27	15.60 ± 0.13	7.30 ± 0.08	1144.55 ± 16.75
LD-YEEs group	6.79 ± 0.35	6.24 ± 0.46	15.90 ± 0.17	7.46 ± 0.14	1163.63 ± 12.97
VC group	7.11 ± 0.59	5.21 ± 0.55	15.54 ± 0.28	7.50 ± 0.22	1158.75 ± 24.17

Note, there was no significant difference among the four groups.

**Table 17:** Effect of YEEs on blood routine of female rats (x ± s).

Group	WBC (10^9^/L)	LYM (10^9^/L)	HGB (10^9^/L)	RBC (10^12^/L)	PLT (10^9^/L)
HD-YEEs group	3.76 ± 0.27	3.48 ± 0.29	15.41 ± 0.18	7.18 ± 0.11	806.75 ± 29.72
MD-YEEs group	4.00 ± 0.36	3.60 ± 0.32	15.13 ± 0.15	7.43 ± 0.11	842.14 ± 42.28
LD-YEEs group	4.27 ± 0.08	3.53 ± 0.29	15.37 ± 0.25	7.09 ± 0.25	794.14 ± 18.29
VC group	3.72 ± 0.25	3.00 ± 0.21	15.33 ± 0.21	7.07 ± 0.12	780.71 ± 80.67

Note, there was no significant difference among the four groups.

**Table 18:** Effect of YEEs on blood biochemistry of male rats (x ± s).

Group	ALT (U/L)	AST (U/L)	ALP (g/L)	Cr (umol/L)	BUN (mg/dL)
HD-YEEs group	120.84 ± 30.99	119.73 ± 38.95	161.86 ± 41.12	53.94 ± 7.89	21.09 ± 2.62
MD-YEEs group	115.20 ± 27.58	115.54 ± 35.91	160.86 ± 38.72	51.62 ± 7.94	19.81 ± 2.72
LD-YEEs group	116.09 ± 29.42	120.73 ± 35.86	162.29 ± 37.37	50.04 ± 8.08	22.40 ± 2.54
VC group	114.96 ± 33.85	119.49 ± 37.17	156.75 ± 39.80	50.89 ± 8.17	20.44 ± 3.25

Note, there was no significant difference among the four groups.

**Table 19:** Effect of YEEs on blood biochemistry of female rats (x ± s).

Group	ALT (U/L)	AST (U/L)	ALP (g/L)	Cr (umol/L)	BUN (mg/dL)
HD-YEEs group	102.38 ± 19.11	129.56 ± 23.82	58.29 ± 13.56	68.30 ± 17.92	27.78 ± 3.43
MD-YEEs group	97.50 ± 22.03	126.12 ± 27.63	51.27 ± 9.12	66.69 ± 16.10	25.35 ± 3.65
LD-YEEs group	97.63 ± 27.86	124.91 ± 27.06	62.83 ± 14.59	67.24 ± 12.03	27.20 ± 3.30
VC group	111.55 ± 19.95	130.34 ± 38.44	60.72 ± 11.48	71.82 ± 11.91	29.05 ± 3.72

Note, there was no significant difference among the four groups.

**Table 20:** Factors and levels for the Box–Behnken experimental design.

Level	(A) Ethanol Concentration (%)	(B) Reflux Time (min)	(C) Liquid–Material Ratio (mL/g)
−1	60	40	20:1
0	70	50	25:1
1	80	60	30:1

## Data Availability

All the data related to this work are included in the article or in the Supplementary Materials.

## Supplementary Materials

The following supporting information can be downloaded at https://www.mdpi.com/article/10.3390/molecules28248124/s1, Table S1: Objective pairwise comparison judgment priority matrix; Table S2: Contents of 13 components (mg/g) and dry extract (%) in single-factor samples; Table S3: Correlation matrix; Table S4: Calculation results of CRITIC of each indicator.
