# Supplementary material for: Optimization of Ethanol Extraction Technology for Yujin Powder Using Response Surface Methodology with a Box–Behnken Design Based on Analytic Hierarchy Process–Criteria Importance through Intercriteria Correlation Weight Analysis and Its Safety Evaluation
Source: Molecules. 2023 Dec 15;28(24):8124. doi: 10.3390/molecules28248124 (PMC10746038; doi:10.3390/molecules28248124)
Supplement: Supplementary file 1 [file molecules-28-08124-s001.zip › Table S1.pdf]

Table S1. 14 Objective pairwise comparison judgment priority matrix

Nemb	Germacrone	Berberine hydrochloride	Baicalin	Baicalein	Wogonoside	Wogonin	Geniposide	chebulinic acid	Paeoniflorin	Emodin	Gallic acid	Chrysophanol	Berberine	Yield of dry extract
**Germacrone**	1	2	2	2	2	2	2	3	3	3	3	3	3	4
**Berberine hydrochloride**	1/2	1	1	1	1	1	1	2	2	2	2	2	2	3
**Baicalin**	1/2	1	1	1	1	1	1	2	2	2	2	2	2	3
**Baicalein**	1/2	1	1	1	1	1	1	2	2	2	2	2	2	3
**Wogonoside**	1/2	1	1	1	1	1	1	2	2	2	2	2	2	3
**Wogonin**	1/2	1	1	1	1	1	1	2	2	2	2	2	2	3
**Geniposide**	1/2	1	1	1	1	1	1	2	2	2	2	2	2	3
**chebulinic acid**	1/3	1/2	1/2	1/2	1/2	1/2	1/2	1	1	1	1	1	1	2
**Paeoniflorin**	1/3	1/2	1/2	1/2	1/2	1/2	1/2	1	1	1	1	1	1	2
**Emodin**	1/3	1/2	1/2	1/2	1/2	1/2	1/2	1	1	1	1	1	1	2
**Gallic acid**	1/3	1/2	1/2	1/2	1/2	1/2	1/2	1	1	1	1	1	1	2
**Chrysophanol**	1/3	1/2	1/2	1/2	1/2	1/2	1/2	1	1	1	1	1	1	2
**Berberine**	1/3	1/2	1/2	1/2	1/2	1/2	1/2	1	1	1	1	1	1	2
**Yield of dry extract**	1/4	1/3	1/3	1/3	1/3	1/3	1/3	1/2	1/2	1/2	1/2	1/2	1/2	1
