# Supplementary material for: Optimization of Ethanol Extraction Technology for Yujin Powder Using Response Surface Methodology with a Box–Behnken Design Based on Analytic Hierarchy Process–Criteria Importance through Intercriteria Correlation Weight Analysis and Its Safety Evaluation
Source: Molecules. 2023 Dec 15;28(24):8124. doi: 10.3390/molecules28248124 (PMC10746038; doi:10.3390/molecules28248124)
Supplement: Supplementary file 1 [file molecules-28-08124-s001.zip › Table S2.pdf]

Table S2. Contents of 13 components (mg/g) and dry extract (%) in single factor samples

Nemb	Germacrone	Gallic acid	Geniposide	Paeoniflorin	Chebulinic acid	Coptisine hydrochloride	Baicalin	Berberine	Wogonoside	Baicalein	Wogonin	Emodin	Chrysophanol	Yield of dry extract
**Ethanol concentration 50%**	2.0087	11.2327	26.5897	74.9702	22.4385	2.8795	15.7169	66.6675	3.6774	4.5143	2.9926	1.6823	22.1451	40.9
**Ethanol concentration 60%**	4.0295	17.2852	41.3008	159.7721	27.8918	2.8001	14.3643	34.8702	4.4738	4.0268	2.3792	2.3959	23.4405	26.85
**Ethanol concentration 70%**	5.7887	13.3970	45.1618	128.2105	21.6098	3.7348	13.4250	32.5532	26.7480	1.3260	2.4717	3.0118	29.3518	28.19
**Ethanol concentration 80%**	3.9806	13.2441	34.7898	108.7280	21.5611	3.4890	11.2888	33.9374	3.6881	1.9777	2.4471	2.5619	26.7725	29.46
**Reflux temperature 40 ℃**	3.4157	7.3506	18.5352	48.4917	21.8474	5.8382	12.9634	55.1385	3.2453	7.4717	3.3815	1.8103	16.7667	23.26
**Reflux temperature 50 ℃**	5.7137	7.3139	21.9865	59.2071	22.5603	6.0375	15.4378	65.3865	4.0131	8.6450	3.3943	1.8154	18.2819	35.49
**Reflux temperature 60 ℃**	6.7145	8.2004	19.9603	55.1335	28.5620	7.0264	21.9377	72.3804	5.2881	9.6080	3.7103	2.3160	19.8155	33.51
**Reflux temperature 70 ℃**	5.5917	7.6257	18.9632	51.0067	27.6846	5.8924	11.4927	65.3091	5.2666	9.2300	3.0999	1.9016	17.8657	31.88
**Reflux time 40 min**	0.6331	6.8493	17.4379	58.8529	23.0539	6.0550	15.2821	71.5981	4.1702	9.5105	3.5784	1.7115	17.2695	35.31
**Reflux time 50 min**	12.9389	7.4851	20.7890	67.7794	25.9481	7.5779	17.9980	77.8741	5.2666	11.7393	3.6719	2.1411	21.0527	32.79
**Reflux time 60 min**	1.5188	9.2642	17.7794	63.5287	23.8216	7.8681	24.3691	85.6418	5.5881	13.8619	3.8442	2.4238	23.1575	32.15

**Reflux time 70 min**	4.3276	6.6537	15.8989	52.2111	21.5793	5.8943	16.4952	70.6266	4.5524	13.1504	3.4337	1.8078	21.7871	36.93
**Ultrasonic intensity 40 KHZ**	1.9442	11.0677	33.7927	60.6240	23.9069	2.6724	15.0352	69.9001	3.0739	10.5744	3.8698	1.8065	15.1298	32.19
**Ultrasonic intensity 50 KHZ**	2.1552	10.3585	33.4649	85.7564	27.2703	7.8140	17.1983	83.4065	7.0737	10.2118	3.3224	2.2793	18.2003	21.6
**Ultrasonic intensity 60 KHZ**	5.4225	6.7026	17.7339	57.4005	23.2245	5.9640	17.6169	62.2914	4.2631	9.4370	3.5183	2.2159	22.1767	23.74
**Ultrasonic intensity 70 KHZ**	4.7600	7.0144	11.0408	48.9522	22.1947	5.3254	17.2198	54.7559	4.1881	8.7049	3.5124	2.2007	20.7280	22.83
**Liquid-material ratio 15:1**	0.3489	4.6301	14.1688	43.6920	19.8672	4.0560	12.3086	46.2230	4.0952	7.1672	3.3539	2.0423	21.6272	36.45
**Liquid-material ratio 20:1**	2.4202	5.7733	17.7794	58.8529	24.2116	6.1285	22.6033	66.8222	5.4059	8.1866	3.5429	2.2996	24.1399	34.93
**Liquid-material ratio 25:1**	6.4913	10.7987	22.2733	131.0974	48.9008	12.8297	37.9808	138.0770	7.4201	19.0138	5.1616	2.2565	21.9985	33.45
**Liquid-material ratio 30:1**	2.1796	7.5768	19.6188	79.9117	36.3064	6.3472	29.5862	101.0440	6.0773	11.3134	3.9515	2.2438	21.9219	32.18
