# Supplementary material for: Optimization of Ethanol Extraction Technology for Yujin Powder Using Response Surface Methodology with a Box–Behnken Design Based on Analytic Hierarchy Process–Criteria Importance through Intercriteria Correlation Weight Analysis and Its Safety Evaluation
Source: Molecules. 2023 Dec 15;28(24):8124. doi: 10.3390/molecules28248124 (PMC10746038; doi:10.3390/molecules28248124)
Supplement: Supplementary file 1 [file molecules-28-08124-s001.zip › Table S3.pdf]

**Table S3.** correlation matrix

| Nemb | Germacrone | Gallic acid | Geniposide | Paeoniflorin | Chebulinic acid | Coptisine hydrochloride | Baicalin | Berberine | Wogonoside | Baicalein | Wogonin | Emodin | Chrysophanol | Yield of dry extract |
|---|---|---|---|---|---|---|---|---|---|---|---|---|---|---|
| **Germacrone** | 1.0000 | 0.0371 | 0.0284 | 0.1251 | 0.2202 | 0.3246 | 0.1035 | 0.0989 | 0.1791 | 0.1505 | 0.0885 | 0.2024 | 0.1195 | 0.0985 |
| **Gallic acid** | 0.0371 | 1.0000 | 0.8886 | 0.8723 | 0.1665 | 0.2884 | 0.0768 | 0.2356 | 0.3273 | 0.4004 | 0.4319 | 0.4597 | 0.3842 | 0.2121 |
| **Geniposide** | 0.0284 | 0.8886 | 1.0000 | 0.7809 | 0.0037 | 0.3904 | 0.2406 | 0.3476 | 0.5265 | 0.5286 | 0.5279 | 0.4960 | 0.3653 | 0.2178 |
| **Paeoniflorin** | 0.1251 | 0.8723 | 0.7809 | 1.0000 | 0.4401 | 0.0197 | 0.1939 | 0.0516 | 0.4466 | 0.2564 | 0.2499 | 0.5931 | 0.5487 | 0.1989 |
| **chebulinic acid** | 0.2202 | 0.1665 | 0.0037 | 0.4401 | 1.0000 | 0.7126 | 0.8427 | 0.8003 | 0.0271 | 0.6132 | 0.6643 | 0.1345 | 0.0232 | 0.0376 |
| **Coptisine hydrochloride** | 0.3246 | 0.2884 | 0.3904 | 0.0197 | 0.7126 | 1.0000 | 0.7711 | 0.8358 | 0.0240 | 0.8192 | 0.7859 | 0.0511 | 0.1301 | 0.0269 |
| **Baicalin** | 0.1035 | 0.0768 | 0.2406 | 0.1939 | 0.8427 | 0.7711 | 1.0000 | 0.8672 | 0.0067 | 0.7261 | 0.8086 | 0.1719 | 0.0918 | 0.1464 |
| **Berberine** | 0.0989 | 0.2356 | 0.3476 | 0.0516 | 0.8003 | 0.8358 | 0.8672 | 1.0000 | 0.1728 | 0.8907 | 0.9026 | 0.1986 | 0.2916 | 0.2215 |
| **Wogonoside** | 0.1791 | 0.3273 | 0.5265 | 0.4466 | 0.0271 | 0.0240 | 0.0067 | 0.1728 | 1.0000 | 0.2975 | 0.2467 | 0.6826 | 0.5806 | 0.1468 |
| **Baicalein** | 0.1505 | 0.4004 | 0.5286 | 0.2564 | 0.6132 | 0.8192 | 0.7261 | 0.8907 | 0.2975 | 1.0000 | 0.9141 | 0.2831 | 0.4048 | 0.1500 |
| **Wogonin** | 0.0885 | 0.4319 | 0.5279 | 0.2499 | 0.6643 | 0.7859 | 0.8086 | 0.9026 | 0.2467 | 0.9141 | 1.0000 | 0.2537 | 0.3794 | 0.1873 |
| **Emodin** | 0.2024 | 0.4597 | 0.4960 | 0.5931 | 0.1345 | 0.0511 | 0.1719 | 0.1986 | 0.6826 | 0.2831 | 0.2537 | 1.0000 | 0.7841 | 0.3940 |
| **Chrysophanol** | 0.1195 | 0.3842 | 0.3653 | 0.5487 | 0.0232 | 0.1301 | 0.0918 | 0.2916 | 0.5806 | 0.4048 | 0.3794 | 0.7841 | 1.0000 | 0.0187 |
| **Yield of dry extract** | 0.0985 | 0.2121 | 0.2178 | 0.1989 | 0.0376 | 0.0269 | 0.1464 | 0.2215 | 0.1468 | 0.1500 | 0.1873 | 0.3940 | 0.0187 | 1.0000 |
