# Supplementary material for: Optimization of Ethanol Extraction Technology for Yujin Powder Using Response Surface Methodology with a Box–Behnken Design Based on Analytic Hierarchy Process–Criteria Importance through Intercriteria Correlation Weight Analysis and Its Safety Evaluation
Source: Molecules. 2023 Dec 15;28(24):8124. doi: 10.3390/molecules28248124 (PMC10746038; doi:10.3390/molecules28248124)
Supplement: Supplementary file 1 [file molecules-28-08124-s001.zip › Table S4.pdf]

Table S4. Calculation results of CRITIC of each indicator

Nemb	Contrast strength δj	Conflict Rij	Comprehensive Weight Ci	Weight Wi
**Germacrone**	0.225	11.421	2.575	0.0711
**Gallic acid**	0.244	11.509	2.812	0.0776
**Geniposide**	0.273	12.164	3.320	0.0916
**Paeoniflorin**	0.284	9.776	2.777	0.0767
**chebulinic acid**	0.227	8.361	1.897	0.0524
**Coptisine hydrochloride**	0.229	9.579	2.198	0.0607
**Baicalin**	0.246	8.588	2.116	0.0584
**Berberine**	0.229	9.681	2.214	0.0611
**Wogonoside**	0.213	11.111	2.369	0.0654
**Baicalein**	0.231	10.907	2.524	0.0697
**Wogonin**	0.220	10.738	2.364	0.0653
**Emodin**	0.249	10.554	2.632	0.0726
**Chrysophanol**	0.240	11.336	2.720	0.0751
**Yield of dry extract**	0.274	13.534	3.710	0.1024
